# Longitudinal Insights Into Pediatric Hypertension, Cardiac Consequences, and Physical Activity

**DOI:** 10.1007/s11906-026-01370-x

**Published:** 2026-03-03

**Authors:** Douglas R. Corsi, Andrew O. Agbaje

**Affiliations:** 1 Department of Medicine, Rutgers Robert Wood Johnson Medical School, New Brunswick New Jersey, US; 2 Institute of Public Health and Clinical Nutrition, School of Medicine, Faculty of Health Sciences, University of Eastern Finland, Kuopio Campus, Yliopistonranta 8, Kuopio, P.O. Box 1627, 70211 Finland; 3 Department of Public Health and Sports Sciences, Childrens Health and Exercise Research Centre, Faculty of Health and Life Sciences, University of Exeter, Exeter, UK

**Keywords:** Temporality, Arterial stiffness, Cardiac remodeling, Physical activity, Sedentary behavior, Cardiac hypertrophy, Preventive Cardiology

## Abstract

**Purpose of Review:**

To summarize important novel but controversial discoveries in pediatric hypertension and prevention. This review also aims to provide some answers to previously unexpected findings in randomized clinical trials that failed to lower blood pressure in youth.

**Recent Findings:**

Emerging longitudinal investigations have elucidated the temporal progression of cardiac structural changes, revealing that arterial stiffness precedes the development of hypertension and cardiac remodeling. These temporal insights have important implications for physical activity interventions, as traditional recommendations emphasizing moderate-to-vigorous physical activity to lower blood pressure have demonstrated limited efficacy in randomized controlled trials. Recent evidence suggests that light physical activity provides superior blood pressure-lowering capacity compared to moderate-to-vigorous physical activity due to the confounding effect of muscle mass. Moreover, sedentary behavior may be a causal risk factor for progressive cardiovascular deterioration.

**Summary:**

Elevated blood pressure and hypertension during childhood and adolescence represent a critical risk factor for premature cardiovascular morbidity and mortality in adulthood. While the association between pediatric hypertension and adverse cardiovascular events is well-established, the temporal sequencing of pathophysiological mechanisms leading from blood pressure elevation to end-organ damage remains unclear. This comprehensive review synthesizes emerging evidence regarding the temporal relationships between pediatric hypertension and cardiac consequences, highlighting temporal trends that could inform evidence-based physical activity interventions for optimizing cardiovascular health trajectories from childhood through young adulthood.

## Introduction

Current epidemiologic data indicate that the prevalence of sustained pediatric hypertension is approximately 4–5% globally, nearly doubling between 2000 and 2020[1], with higher rates observed in populations with increased obesity and among certain racial and ethnic groups[2]. The definition of pediatric hypertension, risk factors, and blood pressure measurement was well explained in a recent review paper [3]. Longitudinal cohort studies consistently demonstrate that elevated blood pressure during childhood and adolescence increases the risk of cardiovascular morbidity and mortality in adulthood [1, 4–13]. Recent population-based data further confirm that children diagnosed with hypertension have a twofold higher risk of major adverse cardiac events in adulthood, including stroke, myocardial infarction, and heart failure, compared to normotensive peers [14].

The pathophysiological mechanisms underlying pediatric hypertension-associated cardiovascular damage remain incompletely characterized, particularly regarding the temporal sequencing of vascular and cardiac adaptations. [15] Traditional recommendations emphasizing blood pressure reduction through moderate-to-vigorous physical activity have led to limited efficacy in randomized controlled trials (RCTs), potentially due to inadequate consideration of confounding variables, including lean body mass and the paradoxical effects of intensive exercise on cardiac structure [16, 17]. A meta-analysis of RCTs in young and middle-aged adults with prehypertension or hypertension concluded that low‐to-middle intensity aerobic exercise was superior to both high‐intensity aerobic exercise and high‐intensity resistance exercise in lowering systolic blood pressure.[18] Recent long-term longitudinal investigations in several thousands of children utilizing accelerometry-based physical activity assessment and cardiac imaging have revealed complex relationships between sedentary behavior, physical activity intensity, body composition and cardiovascular adaptations that challenge conventional exercise prescription recommendations [19].

This comprehensive review synthesizes emerging longitudinal evidence regarding the temporal relationships between pediatric hypertension, cardiac consequences, and physical activity interventions. Understanding these complex interactions is essential for developing evidence-based intervention strategies that optimize cardiovascular health trajectories from childhood through adulthood.

## Cardiac Consequences of Elevated Blood Pressure in Pediatric Populations

### Temporal Progression of Cardiac Structural Changes

Longitudinal investigations across the globe have consistently shown that elevated blood pressure in adolescence leads to progressive cardiac structural and functional adaptations, which persist into young adulthood (Table 1). A 7-year prospective analysis of 1,856 British adolescents revealed that elevated systolic blood pressure and hypertension of ≥ 130 mmHg was associated with 61% increased odds (OR 1.61, CI 1.43–1.80) of progressive left ventricular hypertrophy in female participants but not in male participants. [5] Elevated diastolic blood pressure/hypertension was associated with the odds of worsening left ventricular hypertrophy in both male [1.71 (1.45–2.02)] and female [1.40 (1.30–1.51)] participants. The prevalence of left ventricular hypertrophy increased from 3.6% at age 17 years to 7.2% at age 24 years [5]. Concurrently, left ventricular diastolic dysfunction, defined as E/A ratio < 1.5, progressively worsened from 11.1% to 16.3% prevalence over the same temporal interval and was associated with both elevated systolic and diastolic blood pressure/hypertension. These sex-specific differences may reflect hormonal influences on cardiac remodeling processes, differential body composition effects, or varied hemodynamic responses to blood pressure elevation during adolescent development. [5] Understanding these patterns is crucial for developing targeted intervention strategies that account for biological sex as a modifier of cardiovascular risk.

**Table 1:** Summary of longitudinal associations of changes in childhood and adolescent blood pressure and cardiac structure and function

Author/Year	Sample size	Age in years	Country	Follow-up duration	Study design	Participants health status at baseline	Predictor	Echocardiography outcome	Outcome measure frequency
Agbaje 2023	1856	17 y	UK	7 years	Prospective	Normal	Elevated systolic and diastolic blood pressure/hypertension	Worsening left ventricular hypertrophy Worsening relative wall thickness Worsening left ventricular diastolic dysfunction (E/A) Worsening left ventricular filling pressure (E/e’)	Repeated twice
Zheng et al. 2023	2430	6–19 y	China	30 years	Prospective	Normal	Worse blood pressure trajectory	Higher left ventricular hypertrophy	Once
de Verteuil et al. 2022	142	11 y	Canada	4 years	Prospective	Kidney transplant patients	Blood pressure reduction post-transplant	Left ventricular end-diastolic dimension normalization at 1.5 years post-transplant. Left ventricular posterior wall dimension normalized at 6.3 years post-transplant.	Repeated twice
Heiskanen et al. 2021	1864	6–18 y	Finland	31 years	Prospective	Normal	Higher childhood blood pressure	No associations with left ventricular mass	Once
Liu et al. 2020	1108	4–19 y	US	38.8 years	Retrospective	Normal	Higher childhood systolic blood pressure	Higher left ventricular mass and lower left ventricular diastolic function (E/A)	Once
Hao et al. 2018	683	5–16 y	US	23 years	Prospective	Normal	Increased childhood systolic blood pressure	Higher left ventricular mass	Once
Laitinen et al. 2017	1331	12–19 y	Finland	25 years	Prospective	Normal	Adolescent ideal cardiovascular health (normal blood pressure, lipids, glucose, and body mass index)	Lower left ventricular mass, left ventricular end-diastolic volume, left ventricular filling pressure (E/e’), and left atrium end-systolic volume	Once
Liang et al. 2014	1259	6–18 y	China	24 years	Prospective	Normal	Elevated blood pressure	Higher left ventricular mass	Once

Among 142 pediatric kidney transplant patients from Canada with a mean (SD) age of 11 (± 4.5) years, and followed up for an average of 4 years, there were significant longitudinal changes in cardiac structure and function in association with blood pressure control. [6] Left ventricular end-diastolic dimension normalization was observed at 1.5 years post-transplant. Left ventricular posterior wall dimension normalized at 6.3 years post-transplant, while left ventricular mass index showed sustained improvement up to 12 years post-transplant. However, pediatric patients with uncontrolled blood pressure had increased left ventricular mass[6]. In two Finnish cohorts (*n* = 321 and *n* = 506), normal blood pressure, which was included as part of the 7 components of ideal cardiovascular metrics proposed by the American Heart Association and measured between ages 12 and 19 years, was associated with echocardiography-measured cardiac indices in adulthood 25 years later. In both cohorts, the ideal cardiovascular metrics were inversely associated with left ventricular mass [7]. Another study from a Finnish cohort of 1864 participants measured blood pressure during age 6–18 years and measured left ventricular mass 31 years later, but reported no relationship between childhood blood pressure and adulthood cardiac remodeling [8].

From the US Bogalusa Heart Study, 1108 youth had repeated assessments of body mass index and blood pressure assessments 4–16 times during a mean follow-up period of 38.8 years, and echocardiographic left ventricular structure and function measurements in adulthood. ^9^ Adult left ventricular mass index was significantly associated with childhood and adulthood body mass index, systolic blood pressure, and their area under the curve values, but systolic blood pressure had no significant relationship with left ventricular ejection fraction (Table 1). Adult left ventricular diastolic function (E/A) ratio was negatively associated with adulthood systolic blood pressure [9]. Another US study, the Georgia Stress and Heart Study, where blood pressure was measured 3–16 times during a 23-year period in 546 participants, a higher childhood to young adulthood blood pressure trajectory was associated with higher left ventricular mass index measured in adulthood [10]. In the Beijing Blood Pressure Cohort Study, 1259 Chinese children and adolescents aged 6–18 years were followed over 24 years, and higher childhood blood pressure was associated with a higher left ventricular mass index measured in adulthood [11].

**Fig. 1:**
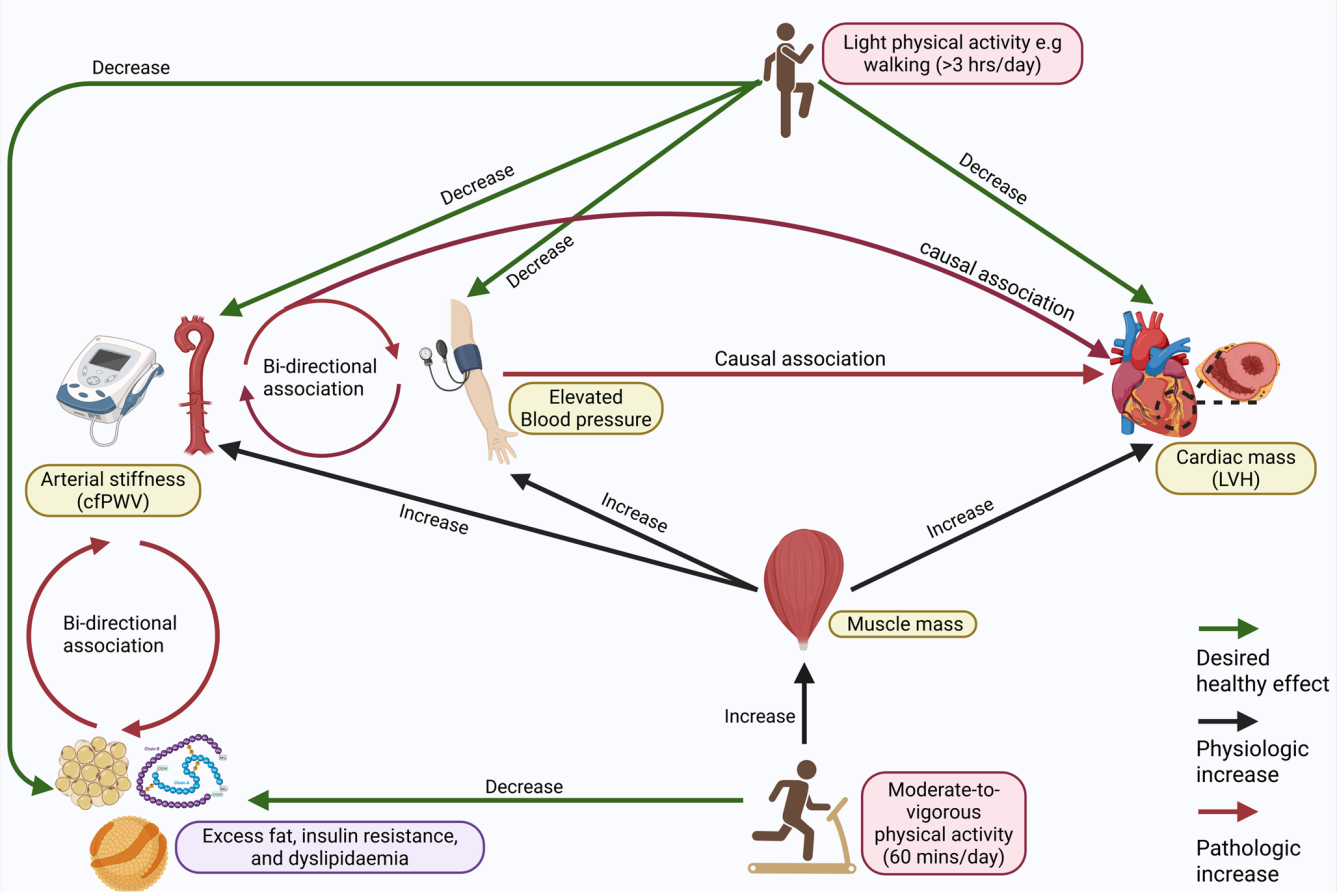
Complex longitudinal and potential causal relationships between the cardiovascular system, body composition, and physical activity in the pediatric and young adult population. cfPWV, carotid-femoral pulse wave velocity; LVH, left ventricular hypertrophy

Additionally, meta-analysis reports concluded that pediatric populations with elevated blood pressure are at risk of significant impairment in global longitudinal strain despite preserved ejection fraction [12]. Furthermore, longitudinal evidence from the Hanzhong Adolescent Hypertension Study in China demonstrates that blood pressure trajectories from childhood to midlife significantly influence cardiovascular damage, with individuals exhibiting persistently high or increasing blood pressure trajectories having elevated risk for left ventricular hypertrophy in midlife (Table 1)[13]. Autoregressive cross-lagged temporal causal path modeling has shown that blood pressure elevation precedes cardiac structural changes, with higher baseline systolic blood pressure significantly associated with subsequent left ventricular diastolic function deterioration [5]. Importantly, baseline cardiac indices had no significant associations with follow-up systolic blood pressure, establishing a unidirectional causality from blood pressure to cardiac damage rather than bidirectional relationships (Fig. 1). Thus, there is consistency across multiple ethnic groups that elevated blood pressure in childhood and adolescence is a causal risk factor for premature cardiac damage, with a pathological remodeling already initiated in the mid-twenties.

### Arterial Stiffness as Temporal Precursor of Elevated Blood Pressure and Cardiac Damage

Adult studies have established arterial stiffness as an independent risk factor for cardiovascular mortality and morbidities. It is known that arterial stiffness worsens with age, but very early arterial stiffening in childhood necessitating interventions is uncommon [20–22]. Previous evidence and clinical pediatric guidelines consistently refer to arterial stiffness (vascular ageing) as an end outcome for risk factors such as elevated blood pressure and hypertension in the young population.[23] Arterial stiffness is often listed as part of target organ damage, a reference to early alterations in vascular physiology before the onset of irreversible pathologies.[24–26] However, emerging evidence among adolescents indicates that arterial stiffness progression temporally precedes cardiac structural damage and development of hypertension (Fig. 1), representing an early biomarker for cardiovascular risk stratification[27]. This suggests that arterial stiffness may not only be considered a vascular outcome to be prevented but also a risk factor for developing cardiometabolic and cardiac pathologies in early life. High carotid-femoral pulse wave velocity progression was significantly associated with worsening left ventricular hypertrophy in a British cohort (*n* = 1856), with particularly pronounced effects in overweight/obese and normotensive subgroups [28]. Among 1171 children from Switzerland, aged 6–8 years and followed up for 4 years, higher blood pressure was bi-directionally associated with higher pulse wave velocity, but the study did not measure left ventricular mass[29]. A prospective longitudinal analysis of 3865 British adolescents reported that higher carotid-femoral pulse wave velocity at age 17 years was significantly associated with elevated systolic blood pressure/hypertension risk and elevated diastolic blood pressure/hypertension risk at age 24 years, establishing arterial stiffness as a temporal precursor to hypertension development rather than a consequence of sustained blood pressure elevation [30]. A cross-lagged temporal analysis demonstrated that higher baseline carotid-femoral pulse wave velocity predicted future left ventricular mass index, relative wall thickness progression, and diastolic function deterioration. Critically, baseline cardiac indices showed no associations with subsequent carotid-femoral pulse wave velocity changes, establishing arterial stiffness as a temporal precursor rather than a consequence of cardiac structural changes [28]. Mediation analyses revealed that cumulative systolic blood pressure elevation accounted for one-third of the direct association between carotid-femoral pulse wave velocity and left ventricular mass progression, while insulin resistance mediated an additional 15.1% of this relationship. These findings suggest that arterial stiffness promotes premature cardiac damage through multiple pathophysiological pathways extending beyond simple hemodynamic effects (Fig. 1).

### Mechanistic Pathways of Cardiac Damage

Increased arterial stiffness augments left ventricular systolic afterload through elevated impedance and reduced arterial compliance, while simultaneously decreasing diastolic perfusion pressure which is essential for coronary circulation[20–22]. This hemodynamic profile promotes compensatory left ventricular hypertrophy as an adaptive response to increased mechanical demands. Additionally, arterial stiffness facilitates inward remodeling of small resistance arteries, creating a pathological feedback loop wherein increased vascular resistance elevates blood pressure, which further accelerates central arterial stiffening[20]. This insidious cycle of progressive cardiovascular deterioration may be particularly detrimental during critical developmental periods (Fig. 1). The microvascular consequences of arterial stiffness extend beyond cardiac effects, with increased pulsatile loads transmitted to vulnerable organ systems, including the brain, pancreas, and kidneys. These effects may establish early foundations for multiorgan cardiovascular and metabolic complications that manifest clinically in later decades [20–22, 27]. Elevated systolic blood pressure-induced cardiac afterload increase also raises intramyocardial wall tension and myocardial oxygen demand.[31, 32] The resulting ventricular hypertrophy impairs coronary flow reserve through capillary rarefaction, perivascular fibrosis, and increased oxygen diffusion distances. [33–35] Additionally, elevated left ventricular systolic pressure increases extravascular compressive forces on intramural vessels, particularly in the sub-endocardium, while arterial stiffening reduces diastolic perfusion pressure.[31, 36] This combination of increased oxygen demand, diminished coronary reserve capacity, and reduced perfusion creates a supply-demand mismatch that predisposes the myocardium to ischemia.[32, 33].

## Physical Activity and Cardiovascular Risk Modification

### Differential Effects of Activity Intensity on Blood Pressure

A RCT conducted among 203 physically inactive young adults (aged 18–35 years) with 24-hour 115/75mmHg-159/99 mmHg blood pressure showed that a 16-week aerobic exercise intervention had no effect on awake systolic (0·0 mmHg [95%CI, −2·9 to 2·8]; *P* = 0·98) or awake diastolic ambulatory blood pressure (0·6 mmHg [95%CI, −1·4. to 2·6]; *P* = 0·58)^16^. It is likely that small samples, short follow-up, adherence/methodological challenges might limit the pooled effect precision, explaining the null findings in RCTs among youth. In addition, the authors did not report the difference in physical activity between the intervention and the controls. A Hawthorne effect is likely due to failed intervention compliance and lack of blinding, which might induce an increase in the control group’s physical activity levels.[16, 37] A meta-analysis of RCTs and quasi-experimental studies synthesized 27 studies, with a sample size of 15,220 children and adolescents aged 6–12-year-old and concluded that physical activity combined with nutrition and behavior change reduced both systolic blood pressure and diastolic blood pressure post intervention (mean difference = −8.64 mmHg and mean difference = −6.75 mmHg), respectively [38]. A meta-analysis of RCTs in adults, including 56 studies from low and middle-income countries, with a total of 10,721 participants, of > 50 years olds concluded that physical activity decreased systolic blood pressure by a weighted mean difference of −7.70 mmHg and diastolic blood pressure by −3.60 mmHg.[39] However, there was a statistically significant high heterogeneity in the studies with diverse physical activity assessments such as aerobic fitness, brisk walking, resistance training, tai chi training, and isometric hand grip training. This variation limits the extrapolation of the meta-analysis report, but the authors emphasised that choosing walking as a physical activity is significantly beneficial for lowering blood pressure. [39].

In another meta-analysis of 19 RCTs including 1590 young and middle-aged (18–65 year old) adults with prehypertension or hypertension, which assessed five exercise training programs concluded that low‐middle intensity aerobic exercise (mean difference − 8.08 mmHg was superior to all exercise strategies (high‐intensity aerobic exercise − 6.53 mmHg; high‐intensity resistance exercise − 4.95 mmHg; compared with the control group in lowering systolic blood pressure. The moderate‐low intensity was defined as 40%–70% maximum heart rate, maximal oxygen consumption or peak power. [18] A recent meta-analysis of 17 RCTs and non-randomized trials involving 1,125 children and adolescents with overweight and obesity, and concluded that 3 months of high-intensity exercise, of three 60-minute sessions per week, reduced systolic blood pressure by a mean difference of −0.44 mmHg and diastolic blood pressure by −0.52mmHg.[40] A meta-analysis of 19 RCTs reported that exercise interventions in children and adolescents with overweight and obesity significantly reduced systolic blood pressure by a standardized mean difference of −0.71 and diastolic blood pressure by −0.67 with an average 3000 min of high-intensity exercise.[41] Another meta-analysis of eight intervention studies concluded that resistance training did not lower systolic and diastolic blood pressure in children and adolescents aged 8–18 years.[42].

Contemporary longitudinal evidence has challenged traditional paradigms emphasizing moderate-to-vigorous physical activity (MVPA) for blood pressure reduction in pediatric populations. A 13-year long prospective analysis of 2,513 children revealed that cumulative accelerometer-measured light physical activity (LPA) was associated with superior blood pressure-lowering benefits than MVPA. Specifically, one-minute cumulative LPA from ages 11 to 24 years was inversely associated with systolic blood pressure (−0.007 mmHg [95% CI −0.009 to −0.004], *P* < 0.001), whereas MVPA showed no statistically significant effects due to absolute confounding by lean mass[17]. Isotemporal substitution analysis revealed that longitudinal replacement of 10 min daily sedentary time with equivalent light physical activity cumulatively decreased systolic blood pressure by −2.63 mmHg (95% CI −3.17 to −2.08, *P* < 0.0001) and diastolic blood pressure by −1.93 mmHg (95% CI −2.36 to −1.50, *P* < 0.0001). On the contrary, equivalent substitution with MVPA yielded no significant blood pressure benefits, highlighting the efficacy of light-intensity interventions [17]. In a recent 6-month long RCT among Finnish adults aged 40–65 years aimed at reducing sedentary behaviour by 1 h/day, an increase in LPA in the intervention group significantly reduced cardiac mass and improved cardiac function when compared to the control group [43].

### Paradoxical Effects of Intensive Physical Activity on Cardiac Structure and Function

A prespecified cardiovascular magnetic resonance imaging RCT sub-study of physically inactive young adults (aged 18–35 years, *n* = 26 intervention and *n* = 32 control) with 24-hour 115/75mmHg-159/99 mmHg blood pressure, concluded that a 16-week aerobic exercise intervention, resulted in decreased left ventricular mass-to-end-diastolic volume ratio, while the right ventricular stroke volume index tended towards an increase for those in the exercise group compared to controls [44]. A recent Coronary Artery Risk Development in Young Adults Study of 1465 US participants with repeated questionnaire-based physical activity spanning a 30-year period and echocardiography assessment in mid-fifties concluded that high physical activity levels were associated with higher left ventricular end‐diastolic dimension index, left ventricular mass index, and left atrial volume index[45]. Among 142 pediatric German athletes aged 14 years, increased aerobic fitness was longitudinally associated with increased left ventricular end-diastolic dimension, interventricular septal thickness, left ventricular posterior wall thickness, and left ventricular mass[46].

In the longest and largest longitudinal study of accelerometer-measured physical activity involving 1682 British children aged 11 years with repeated echocardiographic assessments in adolescence and young adulthood, increased MVPA was associated with a 5% 7-year increase in left ventricular mass[19]. The study highlighted an increased chamber dilatation, left ventricular relaxation, and increasing cardiac mass, reflecting a gradual transition from concentric remodeling to concentric hypertrophy. Nonetheless, the MVPA increase was considered physiological compared to the 40% sedentary behaviour-induced pathological cardiac mass enlargement[19]. In another study of the same British cohort, persistent exposure to ≥ 60 min daily of MVPA in 1339 children aged 11 years and followed up until age 24 years was longitudinally associated with increased carotid-femoral pulse wave velocity progression in both males and females, indicating accelerated arterial stiffening despite presumed cardiovascular benefits[47]. On the contrary, MVPA exposure demonstrated beneficial effects on carotid intima-media thickness progression in females (−0.017 mm [95% CI −0.026 to −0.009], *P* < 0.001), suggesting differential vascular responses between elastic and muscular arterial segments[47]. A systematic review of 16 studies highlighted that continuous light aerobic and resistance exercises may lower peripheral arterial stiffness, likely due to short-term changes in nitric oxide availability, sympathetic nervous system activity, and vascular tone. On the other hand, exposure to chronic high-intensity continuous exercise promotes vascular remodeling of central arteries, through alteration in elastin-collagen ratios.[48] An RCT in middle-aged adults concluded that decreasing sedentary behaviour in the workplace and increasing standing were associated with worsening arterial stiffness.[49] Taken together, exercise-induced cardiovascular remodeling occurs in the young population, and it is important to identify early when physiological adaptation deviates into pathological alterations[50, 51].

There is limited evidence on whether there are any differences in the pathophysiology or long-term outcomes in the cardiac adaptations induced by MVPA in relation to conventional risk factors, such as excess adiposity, dysglycemia or dyslipidemia long-term burden, since these factors are independent risk factors for left ventricular hypertrophy in the pediatric population.[52, 53] MVPA increased left ventricular mass by 5% during 7 years of growth from adolescence to young adulthood.[19] This is significantly less than the independent and combined contributions of lipids, elevated blood pressure, insulin resistance, sedentary time and excess adiposity to increased left ventricular mass, which is > 60%.^5,52,53^ Thus, MVPA-induced cardiac hypertrophy seems physiologic, but conventional risk factors may exert pathological effects on the heart. LPA seems to reverse the pathologic worsening of left ventricular hypertrophy by 49% directly and indirectly by decreasing the adverse effects of conventional risk factors.[5, 19, 52–56].

### Sedentary Behavior as an Independent Risk Factor for Blood Pressure and Cardiac Remodeling

Long term prospective evidence on the association of device-measured sedentary behaviour with increased blood pressure in the young population is scarce, and findings among adults are conflicting [57]. A 3-month parallel-arm randomized clinical trial of 271 US desk workers, aged 18 to 65 years, to increase physical activity decreased sedentary behaviour and increased standing by ≈ 1 h during the workday, but had no effect on blood pressure[49]. In a 6-month randomized controlled trial among Finnish adults aged 40–65 years aimed at reducing sedentary behaviour by 1 h/day, the reduction of sedentary behaviour in the intervention group (*n* = 33) had no effect on cardiac structure and function when compared to the control group (*n* = 31)^43^. A Norwegian study examined 731 children aged 9 years who were followed up until age 24 years, and reported that accelerometer-measured increased sedentary behavior was not longitudinally associated with systolic and diastolic blood pressure[58]. In a Finnish cohort of 153 adolescents aged 6–8 years followed-up for 8 years, sedentary behavior was not associated with impedance cardiography-assessed cardiac work computed from mean arterial pressure, pulmonary artery occlusion pressure, and cardiac index after accounting for adiposity, likely due to a smaller sample size, lack of echocardiography assessment and a gold standard measurement of fat mass[59]. A large-scale longitudinal study recently showed that each one-minute cumulative increase in accelerometer-measured sedentary time from ages 11 to 24 years was positively associated with systolic blood pressure elevation in 2513 British children and progressive left ventricular mass increase in 1682 British children [17, 19]. Notably, sedentary time contributed 40% to the total 7-year increase in cardiac mass (+ 1.29 g/m^2.7^ out of 3 g/m^2.7^ total increase), representing an eight-fold greater contribution than moderate-to-vigorous physical activity (5% contribution)^19^. These findings are independent of the confounding effects of adiposity, tobacco smoking, and hyperglycemia, which are causal risk factors for premature cardiac damage in youth and support sedentary behavior reduction as potentially more important than exercise promotion for pediatric cardiovascular health [53, 60]. The pathophysiological mechanisms linking sedentary behavior to cardiovascular deterioration include increased inflammatory marker expression, impaired glucose metabolism, reduced endothelial function, and adverse effects on autonomic nervous system regulation. These metabolic consequences may be particularly detrimental during critical developmental periods when cardiovascular risk factor clustering establishes adult disease patterns [57].

## Confounding Role of Muscle Mass in Cardiovascular Adaptations

### Lean Mass as Mediator of Exercise-Blood Pressure Relationships 

Muscle growth adapts to an individual’s increase in resistance exercise and nutrition [61]. A 2.6% increase in global muscle mass in humans has been associated with several metabolic improvements, such as lower fat mass, hemoglobin A1c, and fasting glucose level reduction; however, the cardiovascular response to increased muscle mass is paradoxical [62, 63]. Several longitudinal studies in children and adolescents have repeatedly shown that dual-energy Xray absorptiometry (DXA)-measured lean mass without bone mass (muscle mass) is a strong independent determinant of increased blood pressure, carotid thickness, arterial stiffening, and left ventricular hypertrophy during growth until young adulthood, largely explained as cardiovascular remodeling [64–68]. A longitudinal study involving 342 US youth from two longitudinal cohorts examined changes in body mass index with blood pressure from age 13 to 24 years and reported that body mass index did not predict blood pressure in a fully adjusted model. However, baseline systolic blood pressure predicted young adult blood pressure. [69] Similarly, prospective evidence identified lean body mass as a critical confounder in the relationships between physical activity and blood pressure progression in pediatric populations (Fig. 1). Lean mass also doubles as a mediator; for example, mediation analyses highlight that increased sedentary behavior and systolic blood pressure were mediated by lean mass [17]. These findings fundamentally challenge traditional interpretations of exercise-blood pressure relationships and might explain why MVPA interventions in the young population have not lowered blood pressure [16, 46].

The confounding effect of lean mass explains the paradoxical absence of blood pressure benefits from MVPA observed in longitudinal studies and randomized clinical trials [17, 49, 70]. While intensive exercise promotes favorable metabolic adaptations, including improved insulin sensitivity and endothelial function and concurrent lean mass increase, specifically increased smooth muscle mass in vascular tissue, may offset these benefits through hemodynamic mechanisms, such as increased cardiac output demands and enhanced peripheral resistance [57, 68]. Standard epidemiological approaches using body mass index as a covariate may be insufficient to account for the complex relationships between muscle mass, physical activity, and cardiovascular outcomes in the pediatric population [65]. While acute exercise promotes endothelial-dependent vasodilation through nitric oxide production, chronic training-induced vascular remodeling may establish a long-term increase in arterial stiffness that persists beyond immediate post-exercise recovery periods. The balance between beneficial endothelial adaptations and potentially detrimental structural remodeling likely depends on exercise intensity, duration, and individual susceptibility factors [57]. Understanding these complex relationships is essential for optimizing exercise prescriptions in the young population to enhance cardiovascular benefits while minimizing potential adverse consequences.

## Clinical Implications and Future Research Directions

### Evidence-Based Exercise Prescription Guidelines

Current prospective evidence supports careful revision of exercise prescription guidelines for pediatric populations to prevent undesirable elevated blood pressure and adverse cardiovascular sequelae. Traditional recommendations emphasizing strict MVPA may be revised to include LPA > 3 h daily and sedentary behavior reduction below 6 h/day^57,71^. Activities meeting these criteria include leisurely walking, light household tasks, recreational activities, and prolonged standing. These recommendations represent a paradigm shift from conventional guidelines in youth, which lack long-term evidence > 5 years and are largely based on cross-sectional studies and a few-week-long clinical trials, emphasizing shorter durations of intensive exercise for blood pressure reduction. In addition, approximately 80% of youth do not meet the World Health Organization’s MVPA recommendation of an average of 60 min/day [72]. Healthcare providers should receive education regarding the differential cardiovascular effects of activity intensity, with emphasis on the superior benefits of light-intensity interventions and potential adverse cardiovascular consequences of excessive vigorous activity (Fig. 1). The established metabolic effects of MVPA may not automatically translate to a direct cardiovascular health benefit, but rather through indirect approaches, such as reducing dyslipidemia and dysglycemia, which are causal risk factors for cardiovascular damage [52, 53, 55, 71, 73]. Emerging evidence among adults has shown that engaging in LPA prevents the risk of incident cardiovascular diseases and cancers, as well as cardiovascular-kidney-metabolic syndrome.[74, 75] Hence, supporting the explicit inclusion of LPA in future physical activity guidelines is highly recommended.

### Cardiovascular Screening and Risk Stratification

Repeated blood pressure measurements in childhood have enhanced predictive capacity for adult cardiovascular risk compared to single observations, with two abnormal childhood blood pressure measurements yielding superior prediction of adult hypertension (area under the curve 0.77 versus 0.70 for a single measurement, *P* < 0.001) [76]. The temporal precedence of arterial stiffness over cardiac structural changes suggests that carotid-femoral pulse wave velocity assessment may enhance cardiovascular risk stratification in pediatric populations [28]. Routine arterial stiffness measurement could identify individuals at increased risk of future cardiac damage, enabling targeted intervention strategies before irreversible structural changes occur. The clinical utility of arterial stiffness as a screening tool is further supported by its associations with future cardiometabolic risk factors, but large-scale studies performed in children to assess the associations of arterial stiffness with future cardiovascular disease/events are warranted[20, 30, 77]. Contemporary review synthesis supports incorporating arterial stiffness measures into routine pediatric clinical practice, with recommendations for establishing age-specific reference values and standardized measurement protocols to facilitate widespread clinical implementation [3, 27, 77]. Implementation of arterial stiffness screening requires standardization of measurement protocols, establishment of pediatric reference values, and validation of risk prediction models incorporating pulse wave velocity data [77].

### Methodological Considerations for Future Research

Future investigations should prioritize longitudinal study designs with extended follow-up periods to capture the long-term consequences of pediatric cardiovascular risk factors. Cross-sectional studies are only hypothesis generating and do not provide information regarding temporal relationships and causality, limiting their utility for informing intervention strategies, while RCTs usually have short durations [3, 77]. Standardization of physical activity assessment methodologies is essential for enabling comparison across studies and populations. Objective accelerometry-based measurements of physical activity, which are mostly cited in this review, should be preferred over self-report instruments, with emphasis on capturing the full spectrum of movement behaviors, including sedentary time, LPA, MVPA, and vigorous activity. Body composition assessment should be incorporated routinely into pediatric cardiovascular research, with DXA representing the preferred methodology when feasible to account for increasing lean mass during growth and maturation. An accurate and cheap surrogate of adiposity (excess fat) in the pediatric population can be universally assessed using the waist circumference-to-height ratio (WHtR). WHtR has a 90% agreement with DXA-measured total fat mass and trunk fat mass and has been validated as a predictor of type 2 diabetes, bone fracture, liver cirrhosis, blood pressure and heart failure in a multiracial young and adult population. WHtR is fat mass specific and has very poor agreement with lean mass, which is useful to overcome the inaccuracies of body mass index. [78–82] Moreover, the recent Lancet Commission consensus statement on redefining obesity has strongly recommended that obesity should not be diagnosed with BMI measures alone but confirmed with WHtR. [83] Validated sex-specific WHtR pediatric categories has been defined as follows: Low fat mass (< 0.40 for both sexes), Normal fat mass (0.40-<0.50 for males; 0.40-<0.51 for females), High fat mass (0.50-<0.53 for males; 0.51-<0.54 for females), and Excess fat mass (≥ 0.53 for males; ≥0.54 for females). [78] Prospective and objectively measured data from low and middle-income countries are limited on this topic and are urgently needed.

## Conclusions

Contemporary large-scale longitudinal evidence of more than 10-year follow-up duration has fundamentally challenged traditional paradigms regarding physical activity, body composition, and cardiovascular health in pediatric populations. RCTs in the young population are often small sample-sized and of short duration, making their meta-analyses summarises inconclusive. LPA appears to provide superior cardiovascular benefits compared to MVPA, while sedentary behaviour emerges as the primary modifiable risk factor for cardiac structural and functional deterioration. Lean body mass serves as a critical confounder in exercise-blood pressure relationships, necessitating enhanced attention to body composition assessment in research and clinical practice. Arterial stiffness temporally precedes cardiac structural changes in the causal path, providing opportunities for early identification and intervention before irreversible cardiac alterations occur. Understanding these relationships is essential for developing targeted non-pharmacological strategies that optimize cardiovascular health trajectories from childhood through adulthood, potentially preventing the substantial morbidity and mortality associated with adult cardiovascular disease.

## Data Availability

No datasets were generated or analysed during the current study.
